# Bridging bioactive metabolites of *Phoenix dactylifera* L. with advanced nanocarrier technologies for mucoadhesive drug delivery

**DOI:** 10.3389/fphar.2026.1748073

**Published:** 2026-03-24

**Authors:** Abdurrahman Helal Ahmed, Avinash Balasaheb Gangurde, Talib Hussain, Arzu Atalay, Onur Bender, Sirajudheen Anwar

**Affiliations:** 1 Department of Pharmaceutics, KBHSS Trusts Institute of Pharmacy, Malegaon, Nashik, Affiliated to Savitribai Phule Pune University, Pune, Maharashtra, India; 2 Department of Pharmacology and Toxicology, College of Pharmacy, University of Hail, Hail, Saudi Arabia; 3 Biotechnology Institute, Ankara University, Ankara, Türkiye

**Keywords:** cytotoxic, date palm, delivery systems, natural products, NDDS, pharmacological effect, Phoenix dactylifera

## Abstract

*Phoenix dactylifera* L. (PD), a monocotyledonous flowering plant from the Arecaceae family, is one of fourteen species in the Phoenix genus and has been cultivated in the Middle East for over 6,000 years. Its fruit is regarded as a complete food due to its rich nutritional and medicinal profile, containing flavonoids, carotenoids, procyanidins, phenolics, sterols, anthocyanins, steroids, fatty acids, proteins, and amino acids. These bioactive metabolites contribute to its therapeutic potential, particularly in anticancer applications. To enhance the effectiveness of such natural metabolites in medical treatments, Nano-Drug Delivery Systems (NDDS) have gained significant attention, offering controlled drug release, increased permeability and retention, prolonged circulation time, and reduced degradation. This review employed a comprehensive literature search across databases such as Web of Science, PubMed, Google Scholar, and Scopus, using keywords like “*Phoenix dactylifera*,” “date palm,” “Ajwa,” “natural products,” “NDDS,” and “Drug Delivery System,” focusing on relevant publications from January 1995 to May 2025. It provides a comprehensive up-to-date overview of the pharmacological properties of PD metabolites and explores various innovative NDDS formulations including phytosomes, liposomes, microspheres, ethosomes, solid lipid nanoparticles, niosomes, proniosomes, dendrimers, and liquid crystals as potential strategies to improve the delivery and efficacy of PD-based therapies. Additionally, the review discusses the advantages and limitations of these delivery systems in the context of developing PD bioactives as a viable anticancer agent.

## 1 Introduction

**FIGURE 1:**
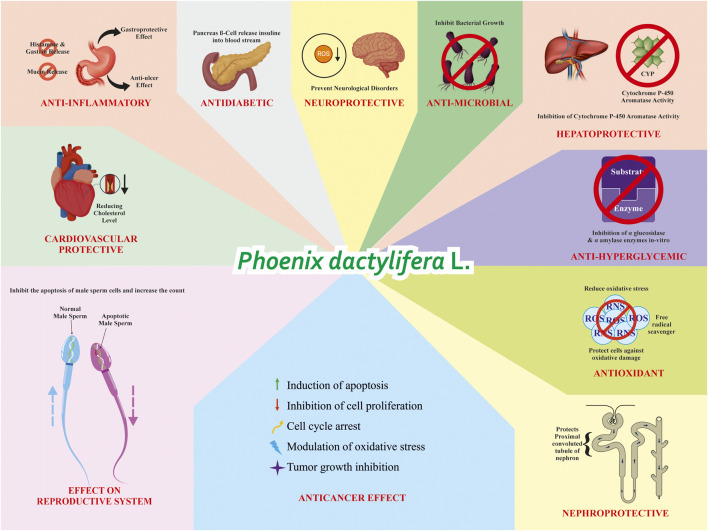
Overview of the therapeutic potential of PD metabolites. This schematic representation illustrates the broad spectrum of biological and pharmacological activities associated with PD metabolites. The fruit exhibits multiple health-promoting effects including anti-inflammatory, antidiabetic, antioxidant, antimicrobial, cytotoxic, antitumor, hepatoprotective, nephroprotective, cardioprotective, anti-hyperglycemic, neuroprotective, and reproductive system-modulating properties. These effects are mediated through various mechanisms such as free radical scavenging, inhibition of key enzymes, modulation of apoptosis, enhancement of insulin secretion, and protective effects on vital organs. Collectively, the figure highlights the therapeutic promise of PD as a multifunctional natural agent in the development of novel pharmaceutical formulations.

Over time, it has become evident that nature contains a vast array of medicinal plants rich in phytochemicals. These plants exhibit strong biological properties, including notable pharmacological effects, and have been utilized for various therapeutic purposes across centuries (Bernardini et al., 2018; Ceylan et al., 2025; Dias et al., 2012; Shen, 2015; Soudi et al., 2024). According to the World Health Organization, approximately 80 percent of the global population relies on traditional medicines. These remedies have been used for centuries to prevent and treat various health conditions. Traditional medicines continue to play a significant role in healthcare systems worldwide (Sirisena et al., 2015). *Phoenix dactylifera* L. (PD) is a flowering plant belonging to the kingdom Plantae, clade Angiosperms, clade Monocots, order Arecales, family Arecaceae, genus Phoenix which comprises approximately 14 species. Among these, PD is the most widely cultivated, especially in the Middle East, where it has been domesticated and used for approximately 6,000 years. Today, it remains an important fruit tree, valued for its nutritional and economic significance*.* (Copley et al., 2001). Phytochemical analysis has shown that PD fruit is a rich source of phenolics, anthocyanins, carotenoids, sterols, flavonoids, and procyanidins. These metabolites contribute to a wide range of biological activities, including antimicrobial, anti-inflammatory, gastroprotective, hepatoprotective, antioxidant, immunostimulant, antimutagenic, cytotoxic, antitumor, antihyperlipidemic, and nephroprotective effects. The diverse pharmacological potential of PD fruit makes it an important natural resource for health-promoting applications (Baliga et al., 2011; El-Far et al., 2016). The bark of PD contains carbohydrates, alkaloids, flavonoids, and tannins. These phytochemicals are associated with various therapeutic effects, highlighting the medicinal potential of the plant. PD bark has thus been traditionally used for health-promoting applications (Hussain Mallhi et al., 2014). Products from the leaves, seeds, pollens, and fruits of PD provide numerous benefits to humans and animals. These health-promoting properties have prompted scientists to explore the plant’s pharmacological potential. Previous studies have shown that supplementing broiler chicken feed with PD seeds enhances antioxidant and immune-stimulating activities (El-Far et al., 2016). Products from PD are commonly consumed as everyday food in Islamic countries, reflecting a tradition that spans over 1,400 years. Historical documents and traditional references frequently mention the consumption of PD, also known as “Ajwa dates” in Arabic, particularly highlighting their reputed health benefits and protective qualities. Classical narrations from early Islamic literature describe regular consumption of PD as providing defense against harmful substances such as toxins or poisons (Al-Bukhari and Sahi, 1976). Dates from the Madina region in Saudi Arabia have long been valued for their therapeutic properties. These dates are commonly consumed and are renowned for their health-promoting effects. Their historical significance highlights the enduring use of Madina dates in traditional medicine. (Siddiqui, 2009). These narratives highlight the ethnopharmacological importance of PD and support its historical use in traditional medicine. They also emphasize the need for scientific investigation into the plant’s bioactive metabolites. Such studies could validate and expand the therapeutic applications of PD. Figure 1 illustrates overview of the therapeutic potential of PD metabolites.

Nanoencapsulation has recently gained considerable attention for delivering bioactive metabolites. This technology offers high loading capacity and entrapment efficiency, while enhancing the stability and bioavailability of the metabolites. It also allows sustained release and can mask undesirable odors or off-flavors, making it a promising approach for improving the effectiveness of natural products (Smaoui et al., 2021). Encapsulation allows the incorporation of plant metabolites such as polyphenols, carotenoids, pigments, fatty acids, phytosterols, probiotics, vitamins, minerals, and bioactive peptides into carrier matrices. These metabolites often have limited stability, and encapsulation enhances their resistance to environmental factors like temperature, light, pH, and oxygen. It also improves their release profile, making them more effective for nutritional and therapeutic applications (Zabot et al., 2022). The incorporation of mucoadhesive polymers such as chitosan, carbopol, hydroxypropyl methylcellulose (HPMC), and natural gums, including date palm mucilage, into nanoparticle systems can markedly enhance therapeutic performance. These polymers promote adhesion to mucosal surfaces, prolonging residence time and facilitating drug absorption across buccal or gastrointestinal membranes. By doing so, they help bypass first-pass metabolism and improve systemic bioavailability (Mansuri et al., 2016; Boddupalli et al., 2010). Several metabolites in PD are either hydrophilic or have high molecular weight, which limits their ability to cross biological membranes. Nanoparticle formulations, especially those containing mucoadhesive polymers like chitosan, can enhance paracellular transport by temporarily modulating mucosal tight junctions. This approach improves the delivery and absorption of PD bioactive metabolites (Bernkop-Schnürch, 2005). In this review, we focus on nanosystem-based strategies as a novel and effective approach for formulating PD. These strategies aim to enhance the delivery and therapeutic potential of PD bioactive metabolites. By leveraging nanotechnology, PD metabolites can be more efficiently utilized for health-promoting applications.

## 2 Methodology

A comprehensive literature search was conducted using multiple databases, including PubMed, Web of Science, Scopus, and Google Scholar, to identify related publications from January 1995 to May 2025. The search employed a combination of keywords such as “*Phoenix dactylifera*” “date palm” “Ajwa” “natural product” “drug delivery” “nanoparticle” “Nano-Drug Delivery Systems (NDDS)” “anticancer” and “bioactivity”. Only peer-reviewed original research articles and review papers published in English were considered. Studies were selected based on their focus on the phytochemical composition, biological activity, and innovative drug delivery formulations related to *P. dactylifera*. Brief reports, opinions and, publications lacking experimental data were excluded. In accordance with best practice standards for phytochemical and pharmacognostic research, all botanical drugs included in this review were evaluated following the Consensus-based reporting guidelines for Phytochemical Characterisation of Medicinal Plant extracts (ConPhyMP; Heinrich et al., 2022). The completed ConPhyMP checklists are provided in the Supplementary Material.

## 3 PD active metabolites

***Phoenix dactylifera*** L. (PD), commonly known as date fruit pulp, is widely recognized for its rich chemical composition and nutritional density. The fruit matrix of PD comprises a diverse range of primary and secondary metabolites, which collectively account for its value as a functional plant-derived food source. These constituents include carbohydrates, amino acids, proteins, fatty acids, vitamins, minerals, and a variety of phytochemicals (Boukouada, 2009; Assirey, 2015; Ali et al., 2014; Boudries et al., 2007). PD is also characterized by the presence of a broad spectrum of secondary plant metabolites. Phytochemical investigations have identified compounds belonging to the phenolics, flavonoids, carotenoids, and steroidal classes (Kchaou et al., 2014; Al-Rimawi and Odeh, 2015; Al-Farsi et al., 2005). These metabolites are structurally diverse and occur in varying concentrations depending on cultivar, ripening stage, and processing conditions. Polyphenolic constituents, including proanthocyanidins and other phenolic derivatives, represent a major fraction of the phytochemical composition of PD (Benmeddour et al., 2013; Vayalil, 2012). In addition to organic compounds, PD contains both water-soluble and fat-soluble vitamins, including vitamins A, C, and E, which are essential micronutrients required for normal physiological maintenance (Vayalil, 2012; Bladé et al., 2016; Al-Shahib and Marshall, 2003). The mineral profile of PD includes essential macro- and micronutrients such as potassium, magnesium, calcium, manganese, copper, zinc, and selenium (Ahmed et al., 1995; Ismail et al., 2006; Al-Hooti et al., 1995). These trace elements are integral components of numerous biological systems and contribute to the overall nutritional quality of the fruit (Adelekan and Thurnham, 1998). Table 1 presents the active metabolites found in different parts of PD.

**TABLE 1:** Bioactive metabolites identified in various parts of PD and their associated pharmacological properties.

S. No	Parts of PD	Metabolites	Therapeutic application (general)	Mode of action	References
1	Pulp	**Sugars:** (mg/100 g m DW) Total sugar-74.3 ± 0.2 Reducing sugar-71.1 ± 0.5 Sucrose-3.2 ± 0.03 Glucose-51.3 Fructose-48.5 ± 0.2	Sweetening agent (Can be used by diabetic patients also)	Binds to the receptor for sweet taste on the tongue in humans	Assirey (2015)
		**Amino acids:** (mg/100 g m DW) Ala-85 Arg-93 Asp-186 Glu-205 Gly-83 His-26 Iso-44 Leu-57 Lys-73 Met-27 Phe-45 Pro-86 Ser- 59 Thr-53 Try-44 Val-65	Immuno- stimulant	T lymphocytes are produced through the binding of Amino acids with the antibodies	Assirey (2015)
		**Minerals:** (mg/100 g m) Mn-0.31 Mg-35.94 Na-7.01 K-290.02 Zn-1.20 P-53.82 Ca-0.339 Fe-0.15 Cd-0.001 Cu-0.37	------	------	Khalid et al. (2017)
		**Phenolic acid:** (mg/100 g m DW) Caffeic acid-0.026 ± 0.001 Ferulic acid-2.52 ± 0.11 Protocatechuic acid-1.217 ± 0.057 Catechin-0.526 ± 0.02 Gallic acid-13.973 ± 0.6 *p*-Coumaric acid- 3.087 ± 0.004 Resorcinol-0.030 ± 0.002 Chlorogenic acid-0.184 ± 0.009 Syringic acid-0.82 ± 0.0 Total Phenolic-22.11 ± 1.10	Anticancer activity Anti-inflammatory Antioxidant	Antitumour Reduce mammary cancer incidence Reduce tumour size and weight Reduce proinflammatory cytokines Inhibits tumour necrosis factor α Decreases ear and paw oedema Enhanced the serum Increasing the level of vitamin C. E Reducing the serum malondialdehyde (MDA)	Hamad et al. (2015) Echega et al. (2020)
		**Flavonoids:** (mg/100 g m DW) Quercetin-1.219 ± 0.071 Luteolin −0.041 ± 0.002 Apigenin-0.263 ± 0.015 Isoquercetin-0.411 ± 0.001 Rutin-0.853 ± 0.049 Total Flavonoid-2.787 ± 0.138	Anticancer activity	Antitumour Reduce mammary cancer incidence Reduce tumour size and weight	Hamad et al. (2015)
2	Seed	**Sugars:** (gm/kg) Glucose-3.5 Fructose-3.8 Stachyose-2.2 Galactose-3.4 Sucrose- 3.5	Hypoglycemic effect Antidiabetic	Increase level of serum c-peptide Control blood sugar level	Alkhoori et al. (2022)
		**Minerals:** (%) Magnesium-0.058–0.090 Calcium-0.014–0.034 Phosphorus-0.110–0.134 Potassium-0.175–0.240 Sodium-0.008–0.013	Metabolic enhancer Cellular energy booster	Generate energy and restore cellular function and growth of Riboflavin	Alkhoori et al. (2022)
		**Amino acids:** (gm/100 g m) Glu-16.44 Phe-5.93 Leu-6.10 Asp-1.72 Ala-1.2 Tyr-1.2 Leu-1.7 Lys-1.1	Immunostimulatory Activity	Amino acids bind with antibodies to produce T lymphocytes	Alkhoori et al. (2022)
		**Total phenolic compound:**109.87–141.72 mg/100 g m	Antidiabetic	Inhibiting enzymes on intestinal glucose absorption	Alkhoori et al. (2022)
		**Total Flavonoids content:** 71.74–86.32 mg/100 g m	Anticancer	Reduction in cancer cell growth	Alkhoori et al. (2022)
3	Leaves	**Total phenolic content:** (Estimated in mg of gallic acid equivalent in 100 g of dry weight)-825.63 ± 0.3	Antioxidant	Scavenging free radicals	Hamini et al. (2025)
		**Total Flavonoids content:** (Estimated in mg of rutine equivalent in 100 g of dry weight)-117.85 ± 0.62	Antimicrobial Anti-inflammatory	------	Hamini et al. (2025)
4	Pollen	**Minerals:** (mg/100gmDW) Ca-1080.00 K-7350.00 Mg-1960.00 B-302.40 Zn-279.90 Se-251 Fe-850.00 Mo-316.90 Cu-365.80 Mn-270.20 Co-198.60 Ni-169.80 Cd-12.00 Na-434	Natural fertility enhancer	↑FSH, LH, and estrogen ↑ Folliculogenesis and ovulation	Chhoud et al. (2025)
		**Vitamins:** (IU/100 g DW) A-7570.50 C-150.70 E−3511.00 (µg/100 g DW) B1-1100–6000 B2-1000–26000 B12-1400-231600	Reproductive health	Balance estrogen and progesterone levels ↑ Endocrine regulation of hormones	Chhoud et al. (2025)
		**Amino acids:** (g/100 g protein) Ile-01.49 Leu-03.34 Lys-02.95 Phe-01.63 Thr-01.72 Val-01.81 His-01.61 Met-00.11 Ala-02.61 Arg-01.61 Asp-03.55 Glu-01.74 Gly-02.24 Ser-01.89 Cys-00.42 Tyr-01.55 Pro-00.28	Hormonal modulation	↑ LH and FSH ↑ Spermatogenesis libido and overall testicular function	Chhoud et al. (2025)
		**Fatty acids:** 7.2%–31.5%	Fertility enhancing effect	Preserve the integrity of reproductive tissues ↑Reproductive health and function ↑ sperm motility	Chhoud et al. (2025)
		**Polyphenols:** 4.96–17.43 mg GAE/g	Antioxidant	Protect spermatozoa from LPO and sperm cells from oxidative stress Protect ovarian tissues from oxidative damage	Chhoud et al. (2025)

## 4 Biological activities of PD

### 4.1 Antioxidant activity

Oxidative stress, primarily induced by reactive oxygen species (ROS) and reactive nitrogen species (RNS), disrupts the body’s antioxidant defense system, resulting in cellular oxidative damage. (Anwar et al., 2014). PD exhibits well documented antioxidant and organ protective activities, primarily attributed to its rich phenolic and vitamin composition, as demonstrated across multiple *in vitro* and *in vivo* studies (Alfaleh and Sindi, 2024; Nawaz et al., 2024). These antioxidant effects contribute to protection against oxidative injury in hepatic, renal, pancreatic and cardiac tissues, underscoring the therapeutic relevance of PD derived metabolites (Nawaz et al., 2024; Awan et al., 2025). However, despite this broad biological potential, the clinical translation of PD antioxidants is constrained by poor aqueous solubility, limited oral bioavailability and rapid metabolic clearance following conventional administration (Khalil et al., 2021; Yuan et al., 2024). Sustained exposure is often required to maintain redox balance and tissue protection, highlighting the need for advanced delivery strategies (Wang et al., 2024). In this context, nanoformulation and mucoadhesive delivery systems offer a compelling approach to enhance stability, prolong mucosal residence time and improve local or systemic bioavailability of PD antioxidants, thereby strengthening their therapeutic efficacy (Wang et al., 2025; Sato et al., 2023).

### 4.2 Cytotoxic, antiproliferative and apoptotic activity

**FIGURE 2:**
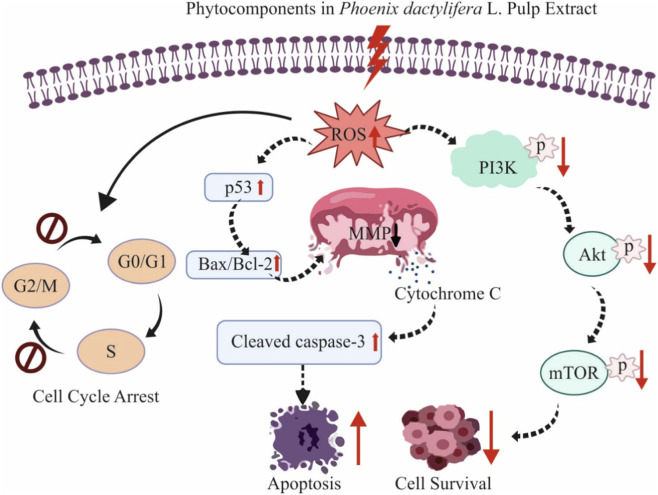
Possible apoptosis mechanism exerted by metabolites in PD pulp extract. This schematic illustrates the molecular pathways influenced by bioactive **metabolites** of PD pulp, leading to anticancer effects. The administration of the extract leads to the generation of intracellular reactive oxygen species (ROS), which disrupts the mitochondrial membrane potential (MMP) and releases the cytochrome c. This cascade enhances the upregulation of p53, alters the Bax/Bcl-2 expression ratio, and triggers the activation of caspase-3, ultimately leading to programmed cell death (apoptosis). Furthermore, there is evidence cellular inhibition through cell cycle arrest. At the same time, the extract downregulates PI3K/Akt/mTOR signaling, contributing to reduced cell survival. These observations indicate a complex anticancer capability mediated through the induction of apoptosis, oxidative stress, and blockade of pro-survival signaling pathways.

Bioactive metabolites derived from various parts of *PD* demonstrate considerable cytotoxic and anticancer activities across multiple *in vitro* and *in vivo* cancer models, including epithelial and hepatocellular malignancies (Abdelkhalek et al., 2024; Al Alawi et al., 2025). These effects, extensively reported in the literature, are generally associated with the induction of apoptosis, disruption of redox homeostasis and suppression of tumor cell proliferation (Al Alawi et al., 2025). Despite this promising anticancer potential, the therapeutic translation of PD metabolites remains limited by poor aqueous solubility, low oral bioavailability and rapid metabolic degradation following conventional administration (Karbasi et al., 2024). Moreover, effective anticancer activity often requires sustained exposure and localized drug tumor interaction, which is difficult to achieve using free extracts or isolated compounds (Lin et al., 2025). These limitations provide a strong rationale for the development of nanoformulation and mucoadhesive delivery systems, which can enhance physicochemical stability, prolong mucosal residence time, enable localized delivery at mucosal tumor sites and improve overall bioavailability, thereby maximizing the anticancer efficacy of PD derived bioactive metabolites (Liu et al., 2025). Figure 2 illustrates the proposed molecular mechanisms underlying the apoptosis and anticancer activity of PD bioactive metabolites.

### 4.3 Anti-inflammatory activity

PD seed and pulp preparations exhibit consistent anti-inflammatory and hepatoprotective activity in experimental models of chemically induced liver injury. In carbon tetrachloride–challenged rats, oral administration of raw or roasted PD seed preparations significantly attenuated hepatic inflammation, fibrosis and tissue degeneration, with roasted seeds demonstrating superior protective efficacy compared to silymarin. Similarly, combined palm date seed and coffee seed extracts markedly reduced inflammatory infiltration and fibrotic changes, effects that were further enhanced upon nano-emulsion formulation (Alqahtani et al., 2023; Abdelaziz and Ali, 2014; Alamri et al., 2024; Khan et al., 2017). Collectively, these findings indicate that the anti-inflammatory and hepatoprotective effects of PD are therapeutically relevant but constrained by conventional oral delivery, highlighting the need for nano-enabled and mucoadhesive systems to improve stability, residence time and bioavailability at target sites (Madbouly et al., 2025).

### 4.4 Other biological activities of PD

PD exhibits a considerable spectrum of biologically relevant activities, including antimicrobial (Allahyari et al., 2021), hepatoprotective (Saafi et al., 2011), nephroprotective (Baothman et al., 2023), neuroprotective (Agbon et al., 2016), reproductive (Mehraban et al., 2014; Omodamiro et al., 2024) and cardioprotective effects (Nawaz et al., 2024), which have been extensively demonstrated in preclinical models. Of particular relevance to drug delivery design, many of these activities rely on phenolics, flavonoids, proanthocyanidins, and lipid-soluble metabolites that suffer from poor aqueous solubility, instability in gastrointestinal environments, rapid metabolism and limited tissue residence following conventional oral administration (Kapare et al., 2024). Antimicrobial and antiviral effects of PD extracts are often concentration dependent and improved by nanoencapsulation, indicating the importance of enhanced stability and local retention (Gomaa et al., 2024; Thirubuvanesvari-Duraivelu et al., 2025). Similarly, systemic protective effects on liver, kidney, brain and cardiovascular tissues largely mediated through antioxidant and anti-inflammatory mechanisms require sustained bioavailability that is difficult to achieve with crude extracts (Nawaz et al., 2024; Al-Nouri et al., 2025; Tomar et al., 2024). Emerging nano based formulations of PD extracts have demonstrated improved biological efficacy compared with conventional preparations, supporting the premise that nano enabled and mucoadhesive delivery systems can enhance physicochemical stability, prolong mucosal residence time and improve both local and systemic bioavailability (Thirubuvanesvari-Duraivelu et al., 2025; Attia et al., 2025). Collectively, these constraints provide a strong rationale for the development of PD based nanoformulations, particularly mucoadhesive systems, to translate its multifunctional bioactivities into reproducible therapeutic outcomes (Thirubuvanesvari-Duraivelu et al., 2025; Lim et al., 2025).

## 5 Novel nano-drug delivery systems (NDDS)

Recent advances in nanomedicine have established ultra-small colloidal carriers such as nanoliposomes, nanosuspensions, micelles, nano-capsules, microemulsions, and solid lipid nanoparticles as versatile platforms to overcome the limitations of conventional formulations. These engineered systems enhance solubility, protect unstable molecules, and improve systemic bioavailability, enabling precise tissue-targeted delivery and expanding therapeutic possibilities (Huang et al., 2024). Nanocarrier systems within the 1–100 nm range including polymeric micelles and solid lipid nanoparticles address the long-standing challenges of poor aqueous solubility and instability in natural products (Huang et al., 2024; Gokul et al., 2024). By stabilizing bioactive metabolites and facilitating efficient absorption, these nano-assemblies substantially amplify the therapeutic efficacy of botanical actives. Furthermore, NDDS enable site-specific transport of natural products, enhancing therapeutic precision and clinical outcomes (Patra et al., 2018). The co-encapsulation of multiple natural bio-actives offers synergistic benefits, where combined pharmacological effects exceed the sum of individual actions (Tadele et al., 2025). Improved delivery efficiency also allows for reduced dosing frequency and enhanced patient compliance (Huang et al., 2024). Collectively, the development of NDDS for natural products represents a rapidly expanding research domain, as previous reviews have primarily focused on single compounds or disease-specific nano-formulation strategies (Huang et al., 2024). Table 2 summarizes all types of nanoparticles.

**TABLE 2:** Summary of various types of nanoparticles utilized in nano drug delivery systems (NDDS).

Types of nanoparticles	Formulation	Application	Advantages	References
Lipid nanoparticles	Solid or liquid lipids stabilized with surfactants (e.g., ionizable cationic lipids, phospholipids, cholesterol)	Gene therapy, RNA vaccines, drug delivery	High biocompatibility, enhanced cellular uptake, controlled release, protection of nucleic acids	Altammar (2023), Hou et al. (2021), Tenchov et al. (2021)
Polymeric nanoparticles	Polymeric like PLGA, chitosan	Cancer therapy, vaccines therapy	Biodegradable, tunable properties	Singh et al. (2023)
Metallic nanoparticles	Metals like gold, silver, iron oxide	Imaging, diagnostics, antimicrobial agents	Unique optical properties, magnetic targeting	Altammar (2023)
Ceramic nanoparticles	Materials like silica, titania	Bone regeneration, drug delivery	Stability, resistance to degradation	Singh et al. (2023)
Quantum dots	Semiconductor materials (e.g.,: CdSe, PbS)	Bioimaging, biosensors	Bright fluorescence, tunable properties	Altammar (2023)
Carbon-based nanoparticles	Graphene, fullerenes, nanotubes	Drug delivery, sensors, electronics	High surface area, conductivity	Singh et al. (2023)
Magnetic nanoparticles	Iron oxide nanoparticles	MRI, targeted drug delivery	Magnetic targeting, responsiveness	Singh et al. (2023)
Dendrimers	Branched polymeric structure	Drug delivery, imaging, cancer therapy	High surface functionality, precision	Singh et al. (2023)
Nanoliposomes	Liposomes and peptides/doxorubicin Liposomes and paclitaxel/carboplatin	Glioma tumor cells Increased MRI (*in vitro*, *in vitro*)	---	Wang and Zhang (2022) Breznica et al. (2020)
Nanoemulsions	Nanoemulsion with fisetin Nanoemulsion with lycopene Nanoemulsion with photosensitizer (e.g., hexylaminolevulinate, aminolevulinic acid)	Lewis lung carcinoma (*in vivo*) Colon cancer (*in vitro*) clinical trials	---	Ragelle et al. (2012) Hu and Zhang (2012) Sell et al. (2023)
Gold nanoparticles	BSA-modified gold nanoparticles	Photothermal therapy (*in vitro*)	---	Al-Jawad et al. (2018)
Iron oxide nanoparticles	Amine-functionalized starch-coated ferrite Nanoparticles with monoclonal antibodies Ferumoxsil, Lumirem® or Gastro MARK®	Breast cancer (clinically approved) MRI scans	---	Korangath et al. (2020) Geppert and Himly (2021)

### 5.1 Merits and demerits of novel drug delivery systems

The development of NDDS has introduced both promising benefits and notable limitations that need to be carefully considered. Consequently, each delivery platform should be evaluated based on its specific characteristics and application requirements (Chen et al., 2024).

- **Liposomal drug delivery system (DDS)** offer several advantages, including low toxicity, excellent biocompatibility, and minimal immunogenicity. However, they also present notable limitations such as low drug-loading efficiency, limited stability, high manufacturing costs, and the potential for dose-dependent toxic side effects (Asadi et al., 2021; Nsairat et al., 2022).
- **Nanoparticle-mediated DDS** are valued for their biodegradability, biocompatibility, and favorable safety and efficacy profiles. Despite these benefits, challenges remain regarding potential nanotoxicity, unclear mechanisms of action, and issues related to polymer degradation and stability (Hsu et al., 2023).
- **Polymeric micelles DDS** exhibit high colloidal stability, enhanced solubilization capacity, and low inherent toxicity. Nevertheless, their application is limited by uncertain long-term safety profiles and restricted clinical translation due to scalability and reproducibility issues (Atanase, 2021).
- **Hydrogel DDS** are recognized for their biocompatibility, biodegradability, and minimal toxic side effects. Their performance, however, is often highly dependent on the internal microenvironment, which can affect drug release kinetics and therapeutic efficacy (Zhang et al., 2024).
- **Inorganic Nanoparticle DDS** demonstrate several advantages, including enhanced bioavailability, low systemic toxicity, and good biological tolerance. However, their broader clinical application is limited by uncertain toxicity profiles, unpredictable biodistribution, and unclear clearance mechanisms within biological systems (Saker et al., 2024).
- **Cell-mediated DDS** offer natural biocompatibility, low toxicity, intrinsic biological functionality, targeting capability, and reduced immunogenicity. Nonetheless, these systems face limitations such as poor control over drug release and restricted drug-loading capacity. (Choi et al., 2023).
- **Extracellular Vesicle DDS** possess desirable features including cycling stability, excellent biocompatibility, and high permeability across biological barriers. However, their development is hindered by immature production technologies and uncertain long-term safety outcomes. (Dżaman and Czerwaty, 2023; Guan et al., 2025).
- **Viral-based DDS** benefit from high transfection efficiency, specific targeting capability, and technological maturity. On the other hand, they suffer from drawbacks such as single-use delivery, strong immunogenicity, safety concerns, and limited payload capacity (Hajebi et al., 2022; Yahoum et al., 2023).

The deployment of nanocarrier systems for *Phoenix dactylifera* anticancer bioactives represents a promising yet evolving frontier in oncological pharmaceutics (Kim et al., 2024). The inherent multifunctionality of PD phytochemicals including antioxidant, pro-apoptotic, and anti-proliferative effects makes them attractive anticancer candidates, yet these benefits are undermined by poor pharmacokinetics and inadequate tumor bioavailability (Bozzuto et al., 2024). While novel nanocarriers such as liposomes, polymeric micelles, solid lipid nanoparticles (SLNs), and nanoemulsions have demonstrated general utility in enhancing drug solubility, stability, and biodistribution, their performance must be critically appraised within the specific context of *P. dactylifera* (PD) anticancer bioactives (Jacob et al., 2025)**.** PD metabolites including phenolic acids, flavonoids, and polyphenols exhibit inherent chemical instability and limited aqueous solubility, which impairs systemic delivery and tumor targeting (Aljabali et al., 2025). Liposomal formulations can enhance the biopharmaceutical profiles of hydrophobic PD components by improving membrane permeability and reducing rapid clearance; however, their clinical translation is hindered by lipid oxidation, vesicle fusion, and unintended immune recognition (Sobol et al., 2025). Polymeric micelles offer controlled release and improved circulation time for PD flavonoids, but polymer degradation products may elicit cytotoxicity and batch-to-batch inconsistency (Cho et al., 2025).

Solid Lipid nanoparticles (SLNs) and Nanostructured Lipid carriers (NLCs) confer high drug loading for lipophilic PD molecules and protection from enzymatic degradation, yet they often experience polymorphic transitions that compromise stability (Alloush and Demiralp, 2025). Nanoemulsions efficiently solubilize poorly water-soluble PD metabolites and facilitate lymphatic uptake, but their thermodynamic instability and propensity for phase separation limit long-term storage. Importantly, active targeting strategies (e.g., ligand-functionalized nanoparticles) can theoretically increase tumor specificity of PD metabolites, but the design complexity and receptor heterogeneity in tumors pose substantial challenges (Cheng et al., 2025; Kumar et al., 2024). Immune recognition and clearance by the mononuclear phagocyte system (MPS) continue to limit effective tumor delivery, underscoring the need for stealth coatings and surface modifications (Lazareva et al., 2025). While each NDDS type offers strategic advantages for enhancing PD anticancer efficacy, limitations related to stability, scalability, immune-compatibility, and targeted delivery must be addressed through rational formulation and preclinical validation (Wu et al., 2025). Collectively, while NDDS technologies hold considerable promise for harnessing PD metabolites as viable anticancer agents, future work must integrate interdisciplinary efforts in materials science, tumor biology, and translational pharmacology to surmount these limitations and realize their clinical potential (Wen et al., 2025).

### 5.2 Recent advances in novel drug delivery systems of herbals

#### 5.2.1 Phytosomes

Phytosomes, also known as phytophospholipid complexes, are an advanced dosage form developed to enhance the stability and therapeutic potential of herbal medicines. Bioactive herbs and their phytochemical constituents are increasingly recognized as valuable therapeutic agents for managing chronic diseases. One of the most effective strategies to improve the efficacy of plant-based therapies involves enhancing the stability and bioavailability of their active compounds. In phytosomes, phospholipids act as carrier molecules that utilize their amphiphilic nature to solubilize, stabilize, and protect herbal extracts. This unique interaction between phytoconstituents and phospholipids not only improves the absorption of lipid-insoluble polar compounds but also significantly enhances their pharmacokinetic and therapeutic profiles. Consequently, both oral and topical phytosomal formulations have demonstrated superior bioavailability and efficacy compared to conventional herbal preparations (Chauhan et al., 2024; Dele et al., 2023; Mardiana et al., 2025).

#### 5.2.2 Liposomes

The term *liposome* is derived from the Greek words *“lipo”* (meaning fat) and *“soma”* (meaning body or structure), referring to a spherical vesicle formed through the self-assembly of amphiphilic molecules, primarily phospholipids. Liposomal delivery systems have emerged as promising platforms to overcome the challenges associated with the *in vivo* delivery of natural bioactive compounds. As versatile and biocompatible nanocarriers, liposomes enhance the solubility, stability, and bioavailability of both natural products and pharmaceutical agents, thereby improving their therapeutic efficacy. Encapsulation of natural compounds within liposomes has been shown to augment their protective effects against cancer, hepatic and neuronal toxicity, inflammation, oxidative stress, hyperlipidemia, and microbial infections. Ongoing research focusing on optimizing liposomal formulations and conducting comprehensive *in vivo* evaluations is essential to better understand their physicochemical stability, targeting mechanisms, cellular uptake, and release kinetics, ultimately advancing the therapeutic potential of natural product-based interventions (Shishir et al., 2019; De Leo et al., 2018).

#### 5.2.3 Microsphere

Microspheres are spherical microparticles, typically ranging from a few to several hundred micrometers in diameter, fabricated from natural or synthetic polymers. They offer significant advantages as drug delivery vehicles, particularly in their capacity to precisely modulate drug release kinetics through controlled adjustments in particle size, surface characteristics, and polymer composition. Owing to their biocompatibility and biodegradability, microspheres ensure safe degradation and clearance from the body following drug administration. Beyond conventional drug delivery, microspheres are being explored as promising platforms for gene transfer and immunomodulation, enabling targeted, sustained, and safe therapeutic outcomes for complex and currently intractable diseases. Furthermore, the integration of microsphere technology with personalized medicine offers substantial potential to enhance therapeutic precision. By tailoring parameters such as polymer type, drug-loading capacity, degradation rate, and surface functionality, microspheres can be customized to align with an individual’s genetic makeup, metabolic rate, and disease progression. Such individualized optimization not only improves treatment efficacy but also enhances patient compliance. Despite their remarkable potential, future advancements in microsphere-based delivery systems will depend on overcoming critical challenges related to large-scale manufacturing, batch-to-batch reproducibility, and precise optimization of drug release kinetics (Yahoum et al., 2023; Lee and Kim, 2025; de Figueiredo et al., 2022).

#### 5.2.4 Ethosomes

The term *ethosome* originates from its principal components‐ethanol and lipids‐, particularly phospholipids. Ethosomes are soft, malleable vesicular systems that exhibit greater deformability than conventional liposomes, enabling them to traverse the narrow intercellular spaces of the stratum corneum, the skin’s outermost barrier. These advanced lipid-based carriers are specifically engineered to enhance transdermal drug delivery. Comprising phospholipids, a high concentration of ethanol, and water, ethosomes facilitate the encapsulation and penetration of both hydrophilic and lipophilic therapeutic agents. The synergistic interaction between ethanol and phospholipids disrupts the lipid organization of the stratum corneum, thereby promoting deeper drug penetration into the skin and, in some cases, systemic absorption. Ethosomes have demonstrated considerable potential in improving the stability, bioavailability, and controlled release of phytochemicals and other bioactive compounds. Recent advancements in ethosomal formulations have focused on encapsulating diverse phytoconstituents, highlighting their physicochemical properties, therapeutic performance, and safety profiles. As an emerging nanocarrier system, ethosomes hold great promise for the transdermal delivery of plant-derived bioactives used in the management of various dermatological disorders. Nevertheless, several challenges remain, including optimization of formulation parameters, large-scale production, and long-term stability, which must be addressed to fully realize their clinical potential (Chauhan et al., 2025; Galatage et al., 2025; El-Menshawe et al., 2019).

#### 5.2.5 Solid lipid nanoparticles

Solid lipid nanoparticles (SLNs) are colloidal drug delivery systems ranging in size from 10 to 1000 nm, developed as effective alternatives to other colloidal carriers such as liposomes, emulsions, and polymeric nanoparticles. These systems can be reproducibly prepared without the use of toxic organic solvents. The solid lipid matrix of SLNs plays a crucial role in improving the stability of incorporated bioactive metabolites. Owing to their composition of biodegradable and biocompatible lipids such as triglycerides, partial glycerides, waxes, steroids, and fatty acids SLNs are considered safe and non-toxic carriers. As an advanced generation of lipid-based nanocarriers, SLNs have gained increasing attention for their potential in delivering a wide range of chemical and natural bioactive metabolites. They offer several key advantages, including enhanced drug stability, improved therapeutic efficacy, and controlled or sustained release profiles. Furthermore, their biocompatibility and environmentally friendly characteristics make SLNs particularly suitable for topical applications targeting the stratum corneum (Shahraki and Daneshmand, 2023; Mostafa et al., 2021).

#### 5.2.6 Niosomes

Niosomes represent a recently developed class of vesicular drug delivery systems with broad therapeutic potential. They consist of bilayer structures formed from amphiphilic non-ionic surfactants and lipid components, primarily cholesterol. Compared to liposomes, niosomes exhibit greater physicochemical stability and can effectively encapsulate both hydrophilic and lipophilic bioactive molecules. These vesicular nanocarriers are biodegradable, relatively non-toxic, stable, and cost-effective, offering a viable alternative to lipid-based systems such as liposomes. Niosomes address several limitations associated with conventional formulations, including poor stability, rapid degradation, low bioavailability, and insolubility of various drugs and natural metabolites. They have demonstrated significant potential in the targeted delivery of therapeutic agents with anticancer, antioxidant, anti-inflammatory, antimicrobial, and antibacterial properties. Moreover, niosomal formulations enable the controlled and sustained release of both hydrophobic and hydrophilic molecules across diverse cancer types. Due to their dual capacity as efficient drug carriers and tumor-targeting systems, niosomes are anticipated to play a pivotal role in the development of next-generation cancer therapies (Liga et al., 2024; Yasamineh et al., 2022).

#### 5.2.7 Proniosomes

Proniosomes are emerging as versatile and efficient drug delivery systems, formulated as dry, free-flowing powders that transform into niosomal dispersions upon hydration. This approach enhances drug stability, solubility, and dosing precision while enabling the delivery of both hydrophilic and lipophilic compounds. Proniosomal formulations can be administered through multiple routes, including oral, topical, transdermal, vaginal, and others, offering broad therapeutic applicability. As a promising alternative to conventional liposomal systems, proniosomes provide controlled and sustained drug release with superior physical and chemical stability. Additionally, their dry, stable nature makes them highly suitable for large-scale manufacturing and commercialization. Proniosomes exhibit excellent potential for improving drug permeation and bioavailability across various delivery routes such as oral, parenteral, dermal, ocular, nasal, pulmonary, and vaginal by overcoming conventional absorption barriers. They can also be formulated into diverse unit dosage forms, including tablets, capsules, and beads. Owing to their versatility, stability, and scalability, proniosomes have attracted considerable research interest as next-generation drug carriers, although further exploration of novel carrier materials and formulations is still warranted to fully realize their therapeutic potential (Ajrin and Anjum, 2022; Eltellawy et al., 2021; Mohanty et al., 2022).

#### 5.2.8 Dendrimers

Dendrimers are three-dimensional, hyperbranched, and monodisperse macromolecules composed of a central core, repeated branching units, and terminal functional groups. Their unique architecture provides abundant internal cavities and modifiable surface functionalities, enabling high structural versatility and biocompatibility. These characteristics make dendrimers ideal candidates for drug delivery applications. Compared to conventional polymers, dendrimers offer several advantages, including high aqueous solubility, multivalency, biocompatibility, and well-defined molecular weights. Various dendrimer types such as polyamidoamine (PAMAM), polylysine (PLL), polypropylene imine (PPI), and polyglycerol (PG) have been widely investigated as delivery systems for natural bioactive metabolites. Dendrimers facilitate targeted drug delivery through multiple administration routes, including intravenous, subcutaneous, intraperitoneal, oral, and ocular pathways. Their nanoscale size, spherical geometry, and multivalent surfaces enable efficient drug loading and release, either through encapsulation or covalent conjugation. By enhancing solubility, bioavailability, and target specificity, dendrimers effectively overcome the limitations associated with many natural products. Future development of dendrimer-based delivery systems should focus on designing multifunctional surface modifications, selecting suitable ligands, and incorporating specific targeting moieties to create tailored systems capable of addressing the structural diversity of natural metabolites and the therapeutic demands of various diseases (An et al., 2023).

#### 5.2.9 Liquid crystals

Liquid crystals (LCs) represent a distinct state of matter that exists between the crystalline solid and isotropic liquid phases. They exhibit unique characteristics, such as the partial or complete loss of positional order of anisotropic constituent molecules, depending on the specific phase. Owing to their exceptional optical and physical properties, LCs have been widely recognized as technologically significant electro-optical materials. While their traditional applications were primarily limited to electronic devices, recent studies have expanded their potential use to the fields of biology and medicine. Notably, emerging research highlights the role of LCs in the identification of cancer biomarkers including nucleic acids, proteins, and enzymes as well as their utility in developing novel diagnostic and therapeutic strategies for cancer. The integration of LCs in cancer detection, prognosis, and treatment demonstrates promising biological implications, particularly in biomarker-based approaches and multi-cell line studies. Future advancements in the application of LCs to cancer biology and modern medicine are expected to be driven by interdisciplinary collaboration among chemists, biologists, physicists, clinicians, material scientists, and engineers (Vallamkondu et al., 2018; Su et al., 2015).

#### 5.2.10 Hydrogels

Hydrogels are soft, hydrophilic polymer networks characterized by high water content, tunable mechanical properties, and excellent biocompatibility. They are defined as three-dimensional polymeric systems capable of efficiently absorbing and retaining water or physiological fluids while maintaining their structural integrity despite dimensional changes. Owing to these unique characteristics, hydrogels have attracted significant interest as biomaterials in modern medicine.

In cancer therapy, hydrogels represent a promising research area aimed at enhancing therapeutic efficacy while minimizing toxicity and adverse effects. A recent PubMed search using the terms “hydrogel” and “oncology” yields approximately 275 publications from the past 5 years, highlighting growing scientific attention. Most current applications of hydrogels in oncology remain at the preclinical stage, including their use as environmentally responsive drug delivery platforms, nanogels for radioisotope delivery, and embolization agents. These systems require further investigation to evaluate their safety, biodistribution, and drug release profiles. Clinically, the use of Gliadel® wafers (hydrogel-based implants delivering carmustine for glioma treatment) demonstrates their translational potential. Hydrogels are also increasingly utilized to model the tumor microenvironment, serving as scaffolds that mimic the extracellular matrix (ECM) for cancer cell growth. Such three-dimensional culture systems more accurately replicate *in vivo* tumor conditions compared to conventional two-dimensional models, providing valuable insights for oncological research. Future directions in hydrogel development focus on designing multifunctional and stimuli-responsive systems capable of enabling personalized and adaptive cancer therapies. Advances in nanotechnology, 3D bioprinting, and bioactive molecule integration are expected to expand their applications in regenerative medicine, organ engineering, and precision oncology. The major challenges ahead include enhancing long-term stability, ensuring consistent biocompatibility under complex physiological conditions, and establishing scalable, cost-effective production methods to facilitate clinical translation. Table 3 describes the role of NDDS in cancer therapy and other diseases (Rybicki et al., 2025; Yan et al., 2025).

**TABLE 3:** Overview of different NDDS types and their reported biomedical applications across various disease conditions.

NDDS	Applications	References
Phytosomes	Antioxidant, anti-inflammatory	Dele et al. (2023)
Liposomes	Cancer, fungal and viral infection	Jaromin et al. (2024), Teixeira et al. (2025), Kocas et al. (2024)
Emulsions	Cytotoxicity, antimicrobial Cardiovascular disease, antidiabetic	Roselan et al. (2020), Tominc et al. (2024), Ananingsih et al. (2024)
Microsphere	Anti-diabetes	Yahoum et al. (2023)
Ethosome	Breast cancer, antifungal	Galatage et al. (2025), Huanbut et al. (2022)
Niosomes	Anti-hyperpigmentation, disease affecting the brain	Kanpipit et al. (2024), Jin et al. (2013)
Proniosomes	Antiulcer, antioxidant	Mohanty et al. (2022), Shaghayegh Alavi et al. (2021)
Dendrimers	Antioxidant activity	Igartúa et al. (2023)
Nanoparticles	Neuroprotective activity	Khorrami et al. (2023)
Liquid Crystals	Anti-inflammatory, antioxidant, antibacterial, anti-tuberculosis, antifungal	Santos et al. (2022), Kósa et al. (2021), dos Santos Ramos et al. (2015)
Hydrogel	Antioxidant, antibacterial, analgesic, anti-inflammatory	Pelin et al. (2023), Abbas et al. (2023)
TDDS	Heart Failure, antidiabetic	Mo et al. (2022), Kaushik and Joshi (2024)

## 6 Delivery platforms for PD in nano-systems

**FIGURE 3:**
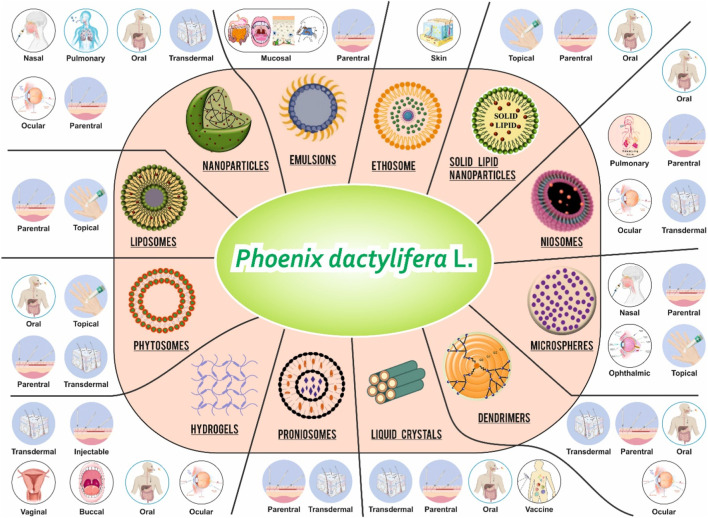
All possible NDDS formulation for PD and its routes of administration. PD, rich in bioactive phytochemicals with reported antioxidant, anti-inflammatory, and hepatoprotective effects, offer potential for incorporation into various advanced drug delivery systems. The central panel illustrates a variety of nanocarriers that can be employed to enhance the bioavailability, targeting, and therapeutic efficacy of PD date-derived metabolites. These include nanoparticles, liposomes, phytosomes, proniosomes, hydrogels, ethosomes, niosomes, microspheres, dendrimers, emulsions, solid lipid nanoparticles, and liquid crystals. Each delivery system is associated with multiple administration routes, including oral, nasal, pulmonary, ocular, topical, transdermal, buccal, vaginal, injectable, mucosal, and parenteral pathways. This conceptual framework underscores the versatility of PD date metabolites for formulation into novel delivery platforms to maximize therapeutic outcomes in various disease models.

As a result of the extensive literature survey, it was discovered that there are fewer formulations stated in review and research articles, indicating the necessity to formulate several nano-systems utilizing different polymers for varied biological activities with the desired benefits. The development and delivery of mucoadhesive anticancer nanoparticles incorporating PD extracts represents an emerging convergence of natural product pharmacology and advanced drug-delivery engineering (Chuah et al., 2014; Gómez-Guillén and Montero, 2021; Saa et al., 2024). Integrating plant-derived anticancer bioactives with nano-enabled mucoadhesive systems offers a promising strategy to overcome intrinsic barriers associated with mucosal drug delivery, enhance drug retention at tumour-associated mucosal sites, and ultimately improve the therapeutic efficacy of botanical metabolites (Popovici et al., 2022). A critical aspect in the design of PD-based mucoadhesive nanoparticles is the rational selection of the polymeric matrix and its functional performance. Cationic biopolymers, particularly chitosan, are frequently employed owing to their excellent biocompatibility and pronounced mucoadhesive properties (Imam et al., 2021). These characteristics are primarily attributed to electrostatic interactions between positively charged amino groups of chitosan and negatively charged mucin, complemented by extensive hydrogen-bonding that prolongs mucosal residence time (Sharifi-Rad et al., 2021). When applied to PD-based nanoformulations, such mucoadhesive systems can substantially enhance both localized and systemic delivery of PD bioactives via buccal, nasal, gastrointestinal, or vaginal routes. Furthermore, the small particle size and strong mucoadhesive behavior of chitosan nanoparticles facilitate effective penetration across biological barriers, targeted drug delivery, and reduced dose-related toxicity (Imam et al., 2021). Notably, higher molecular weight and degree of deacetylation of chitosan are associated with enhanced mucoadhesive strength (Alshehr et al., 2020). These physicochemical attributes also contribute to improved encapsulation efficiency by stabilizing flavonoid-rich PD extracts within the nanoparticulate matrix, thereby supporting sustained and efficient drug delivery (Davodabadi et al., 2025). Some of the formulations of PD cited in the research articles are mentioned below. Figure 3 and Table 4 describe NDDS of PD metabolites.

**TABLE 4:** Overview of PD-based formulations developed using different NDDS approaches.

PD formulations	Polymers	Particle size	Encapsulation efficiency	Pharmacological outcomes	Applications	References
Nano-emulsions	Oleic acid Tween 80 PEG	∼23.3 nm (spherical morphology) (TEM) ∼69.9 ± 27.9 nm (DLS)	Not quantitatively reported; nanoemulsions protected bioactives and maintained antioxidant activity via sustained release	Significant improvement in liver antioxidant enzymes (SOD, CAT, GSH, GPx) vs. CCl4 fibrosis model; reduced MDA and inflammatory markers	Liver Fibrosis	Alamri et al. (2024)
R and Q Nanoparticles	PLGA DSPE-PEG	∼200 nm average diameter (measured by dynamic light scattering)	∼70% entrapment efficiency for RQ-NPs	DOX alone reduced tumor volume and weight significantly; DOX + RQ-NPs also reduced tumor growth without significant interference from RQ-NPs alone	Anti-cancer	Godugu et al. (2020)
Silver nanoparticles (AgNPs)	Silver nitrate	Spherical nanoparticles with diameters **15–80 nm** (TEM)	Not reported	Dose-dependent cytotoxicity against human breast cancer cell line HCC712: increasing AgNP concentration (25–500 μg/mL) decreased cell viability, with significant effects at ≥100 μg/mL Significant antibacterial activity against Gram-negative and Gram-positive bacteria (e.g., *Klebsiella pneumoniae*, *Pseudomonas aeruginosa*, *Escherichia coli*, *Staphylococcus aureus*, *Enterococcus faecalis*) with inhibition zones measured at ∼17–20 mm at 100 μg/mL Inhibition of bacterial biofilm formation: e.g., ∼70% against *E. coli*, ∼66% against *S. aureus*, and lower inhibition (∼39–43%) against other strains at tested concentrations	Cytotoxic Anti-bacterial Anti-biofilm	Allemailem et al. (2022)
Nanoemulsion	PEG/surfactant system for stabilization	∼115–235 nm (varied with formulation)	Not explicitly stated (optimized for stability & release)	Extended *in vitro* release; significant cytotoxicity against MCF-7 and HepG2 cancer cells (IC50 ∼18.6 & 13.5 μg/mL respectively) — indicating anticancer potential	Anticancer	Khalil et al. (2021)
Hydrogel	HPMC hydrogel base	DP-NLC: ∼266.9 nm	∼77.9% *in vitro* release from NLC; ∼42.4% from hydrogel matrix (functional release)	(*Bacillus*, *S. aureus*, *K. pneumoniae*); good hydrogel physical properties & stability	Topical antibacterial	Elsewedy et al. (2023)
O/W nanoemulsion	Surfactants (aqueous titration)	∼23.1 nm	∼99.7% drug content	delivery and improved extract release profile — model for inflammatory therapy	Antioxidant	Qadir et al. (2020)
Ag/AgCl Silver nanoparticles	Plant phytochemicals act as stabilizing agent	165.5 nm	73.57% for *P. aeruginosa* and 88.66% for *S. aureus* at 1*MIC, respectively	Inhibition of bacterial biofilm formation, especially in reducing biofilm development	Antibacterial antibiofilm	Thirubuvanesvari-Duraivelu et al. (2025)
Silver nanoparticles	Date palm mucilage (polymeric excipient)	139.7 nm	NA (composite)	Behavior in colonic delivery models; mucilage provides polymeric carrier	Sustained release	Shahid et al. (2021)

## 7 Future prospects, opportunities, obstacles and challenges

The application of NDDS in PD represents an exciting avenue in the fields of nutraceuticals, pharmaceuticals, and functional foods. PD bioactive are renowned for their medicinal and nutritional benefits due to their rich content of bioactive metabolites like phenolics, flavonoids, dietary fiber, and antioxidants. Leveraging NDDS for PD metabolites can help enhance their bioavailability, stability, and targeted delivery. There are some key prospects and opportunities like encapsulation of bioactive metabolites, improved absorption, incorporation into functional foods and beverages, pharmaceutical and nutraceutical formulations, topical applications, as well as expanded opportunities for research, clinical translation, and market growth. The integration of NDDS with PD offers a transformative opportunity to harness their full therapeutic and nutritional potential. Continued research and development, supported by interdisciplinary and industrial collaboration scan, may lead to the development of innovative, effective, and commercially viable products. PD dates include a variety of bioactive compounds, such as flavonoids, polyphenols, glycosides, tannins, and others. The identical active component may be difficult to extract, standardize, and disseminate. Additionally, processing, location, and season can all affect the phytochemical composition. Many of the bioactive ingredients in PD dates, including certain flavonoids, have limited water solubility; in particular, NDDS that is aqueous-based exhibits poor bioavailability. Solubility-enhancing techniques (such as the use of solid dispersions or surfactants) are required. Since PD dates are nutraceuticals rather than single-molecule APIs, regulatory obstacles for NDDS applications could arise. Pharmacokinetic and pharmacodynamic studies are more difficult when dosage cannot be standardized. For NDDS to be effective, pure, concentrated active ingredients are usually required. One disadvantage is that the extraction procedure from PD dates can be laborious and solvent intensive. Processing-related degradation risk, some PD date constituents may destabilize or adversely interact with synthetic carriers.

An emerging frontier in the delivery of *PD* metabolites lies in the integration of mucoadhesive polymers with current nano-drug delivery systems to enhance mucosal residence time, absorption and therapeutic efficacy (Zheng et al., 2025). Mucoadhesive polymers such as chitosan and its chemically modified derivatives have demonstrated significant potential for transmucosal drug delivery due to their ability to interact electrostatically and via hydrogen bonding with mucosal glycoproteins, thereby prolonging contact with mucosal surfaces and facilitating sustained release and absorption of encapsulated therapeutics (Elella and Kolawole, 2024). Chitosan possesses intrinsic biocompatibility, biodegradability and mucoadhesive properties, which contribute to prolonged gastric and buccal retention, enhanced permeation across epithelial barriers and protection of labile compounds from enzymatic degradation (Saputra and Andreas, 2025). Chemical modifications of chitosan (e.g., trimethylation, thiolation) can further improve mucoadhesion and mucosal permeation beyond that of native chitosan, offering tailored platforms for optimized plant derived metabolite delivery through buccal, gastrointestinal, or nasal routes. Similarly, carbopol exhibits excellent mucoadhesive strength, pseudoplastic flow behavior, compatibility with mucosal pH ranges, and can prolong formulation residence time, enabling controlled release and enhanced mucosal uptake of bioactive agents (Elella and Kolawole, 2024; George et al., 2025).

Date palm mucilage, a natural plant derived polymer, represents a biocompatible excipient with intrinsic mucoadhesive potential that could be explored both as a standalone platform or in combination with nanoparticles, hydrogels, or liposomes to improve PD metabolite stability and localization at mucosal tissues (Shahid et al., 2021; Mondal et al., 2025). The strategic incorporation of these mucoadhesive polymers into NDDS such as mucoadhesive nanoparticles, films, gels, or patches has the potential to overcome physiological clearance mechanisms, enhance mucosal penetration, and increase systemic absorption of *PD* metabolites, thereby broadening their translational relevance in therapeutic applications (Dubashynskaya et al., 2024). Future research should focus on *in vivo* validation, optimization of polymer drug compatibility, and translational evaluation across diverse mucosal sites to fully harness this promising mucoadhesive NDDS approach.

The translational development of PD–based nanocarrier systems holds considerable promise but is limited by interrelated challenges encompassing extract standardization, formulation stability, regulatory classification, long-term safety, and scalable manufacturing. PD extracts display substantial phytochemical heterogeneity arising from cultivar, geographic origin, maturity stage and extraction protocols, which can significantly influence nanoparticle formation, physicochemical properties and biological performance, thereby undermining quality control and regulatory confidence (Parvin et al., 2025; Andreani et al., 2024; Rahat et al., 2024). To mitigate this variability, advanced chemoprofiling approaches particularly LC–HRMS-based metabolic fingerprinting integrated with chemometric modeling within a Quality by Design (QbD) framework are increasingly recommended to ensure batch-to-batch reproducibility and translational robustness (Olivaro et al., 2025; El Boudlali et al., 2025).

Formulation stability represents an additional critical barrier, as nanocarriers are inherently susceptible to aggregation, fusion, and structural rearrangements during storage and transport, potentially altering drug loading, release kinetics, and bioavailability (Singh et al., 2025). Although stabilization strategies such as surface functionalization and lyophilization have shown promise, their applicability and long-term effectiveness must be systematically validated for specific PD-derived payloads and carrier architectures (Rahat et al., 2024; Ruppl et al., 2025).

Regulatory uncertainty further complicates clinical translation, as PD extracts traditionally positioned as nutraceuticals may be reclassified as drugs or nanomedicines when engineered for therapeutic intent, systemic exposure, or targeted delivery. While nanotechnology does not intrinsically mandate reclassification, it often triggers more stringent requirements for safety, quality, and efficacy evaluation (Rodríguez-Gómez et al., 2025; Mangla et al., 2025). Importantly, although PD bioactives are generally regarded as safe in conventional formulations, nanoencapsulation can modify pharmacokinetics, biodistribution, and cellular interactions, raising potential concerns regarding long-term safety (Oladipupo et al., 2025). Enhanced mucosal penetration and prolonged residence time—may simultaneously increase off-target exposure and immune engagement, underscoring the need for extended *in vivo* safety assessments beyond acute and subacute models (Zheng et al., 2025; Boushehri, 2025).

Finally, scale-up and manufacturing remain major obstacles, particularly for green and extract-mediated synthesis strategies that often lack industrial robustness. The ability to maintain critical quality attributes in the absence of integrated process analytical technologies (PATs) constitutes a key bottleneck for commercialization and clinical deployment (Aguiam et al., 2025; Ansari et al., 2024; Abegunde et al., 2024).
